# Response to letter regarding “Gastrointestinal foreign bodies in pet pigs: 17 cases”

**DOI:** 10.1111/jvim.16478

**Published:** 2022-07-08

**Authors:** Yoko Nakamae, Diego E. Gomez, Kallie J. Hobbs, Jessie Ziegler, Luis A. Rivero, Shari Kennedy, Jenna Stockler

**Affiliations:** ^1^ Department of Clinical Studies, Ontario Veterinary College University of Guelph Guelph Ontario Canada; ^2^ College of Veterinary Medicine University of Florida Gainesville Florida USA; ^3^ School of Veterinary Medicine University of California ‐ Davis Davis California USA; ^4^ College of Veterinary Medicine University of Missouri Columbia Missouri USA; ^5^ College of Veterinary Medicine Auburn University Auburn Alabama USA

Dear Dr DiBartola and Dr Hinchcliff,

We would like to start by thanking Dr Smith et al for their letter highlighting a discrepancy in our article.^1^ This article should have clearly stated that some pigs received antimicrobial drugs off‐label to align with JVIM's antimicrobial language policy.^2^

Off‐label use of antimicrobial drugs can be defined as the administration of an antimicrobial drug for indications not approved by regulatory authorities, or at a different dose or frequency, or to different age groups, or by administering through a different route.^3^ All the antimicrobials used in the pigs enrolled in our study are legal and can be used in an extralabel manner in pigs in the United States and Canada under a valid veterinary‐client‐patient relationship. As indicated by Smith et al, we should have clearly stated that some antimicrobial drugs administered to these pigs were used in an extralabel manner and that in all our academic institutions the veterinary‐client‐patient relationship includes recommendations regarding strict adherence to the withdrawal times.

As mentioned by Smith et al, none of the drugs used in the pigs enrolled in this study are labeled for surgical prophylaxis or similar indications in the United States. Many of the pigs included in this study were at a high risk of respiratory disease associated with vomiting (13/17). Furthermore, 83% (14/17) of the pigs were initially treated medically but failed to respond and an exploratory laparotomy was performed.^1^ Therefore, it is likely that in many cases initial antimicrobial therapy was instituted for treatment of respiratory disease rather than surgical prophylaxis. However, due to the retrospective nature of this study it is challenging to determine the clinician decision‐making process for antimicrobial drug administration in each pig.

Pet pigs present a challenge to veterinarians regarding antimicrobial drug selection because diseases affecting pet pigs can be considerably different to those commonly observed in production animals. Therefore, current label recommendations of antimicrobials for food animal use might not apply when treating common infectious diseases in pet pigs, which would often lead to off‐label usage. We agree with Smith et al, that despite the differences in animal use and the nature of the veterinary‐client‐patient relationship between production and pet pigs, veterinary practitioners must adhere to the current antimicrobial drug use recommendations for swine.

The authors of this study appreciate the excellent point highlighted by Smith et al, and we commend them for their continued interest in expanding the current body of literature in pet pigs.

Sincerely,
